# Tenascin-C serum levels and its prognostic power in non-small cell lung cancer

**DOI:** 10.18632/oncotarget.7976

**Published:** 2016-03-08

**Authors:** Florian Gebauer, Suyin Gelis, Hilke Zander, Karl-Frederick Meyer, Gerrit Wolters-Eisfeld, Jakob R. Izbicki, Maximilian Bockhorn, Michael Tachezy

**Affiliations:** ^1^ Department of General, Visceral and Thoracic Surgery, University Medical Center Hamburg-Eppendorf, Hamburg, Germany

**Keywords:** lung cancer, NSCLC, prognostic marker, biomarker, serum

## Abstract

**Background:**

Tenascin-C is overexpressed in the stroma of most solid malignancies and may function as a diagnostic tumor marker. This study was conducted to evaluate the potential significance of Tenascin-C as a predictive marker for tumor progression in the sera of non-small cell lung cancer (NSCLC) patients.

**Results:**

Serum concentration of Tenascin-C is significantly elevated in NSCLC patients compared to healthy controls (p=0.013). The sensitivity of Tenascin-C in detecting NSCLC was 74% at a specificity of 57%. Elevated Tenascin-C serum values are associated with larger tumor size and lymph node involvement (p=0.022 and p=0.036, respectively). The Kaplan-Meyer-curves showed a significant association of Tenascin-C with the patient's overall survival (p=0.004), but not with the recurrence-free survival (p=0.328).

**Methods:**

We quantified Tenascin-C in the sera of 103 NSCLC patients and 76 healthy blood donors by enzyme-linked immune-absorbance assay tests. Prognostic significance was determined by area under the curve analysis and Youden-index. The results were correlated with clinical, histopathological, and patient survival data (Chi-square test, Kaplan-Meier analysis, log-rank test, multivariate Cox-regression analysis).

**Conclusion:**

Although significantly elevated in patients with NSCLC, the sensitivity and specificity of the Tenascin-C serum quantification test was low. However, although failing to be an independent prognosticator in multivariate analysis, the results implicate Tenascin-C as a predictive prognostic marker for NSCLC patients. The data must be further validated in future prospective trials with larger patient cohorts.

## INTRODUCTION

Due to its aggressive biology, lung cancer causes the most cancer-related deaths worldwide [1]. Non-small-cell-lung cancer (NSCLC) accounts for 85% of all lung cancers with a high metastatic potential and a high rate of tumor recurrence, even after curative intended tumor resection. The prognosis for patients with NSCLC depends mainly on classical clinical aspects such as tumor size, grading and the existence of metastases; clinical decision-making is still based on the classical UICC staging system [2]. Although significant improvements of the individualized therapy for NSCLC patients have been made during the last few years with introduction of several molecular markers (such as EGFR and EML4-Alk) in the clinical routine, the search for a clinically relevant serum marker as diagnostic and prognostic markers is still ongoing [3]. Tumor biomarkers with prognostic power regarding treatment success or prognostic impact on survival might significantly help to stratify the therapy of patients and improve the diagnosis and therapy.

In the case of NSCLC, known biomarkers such as SCCA, CYFRA and CEA show low sensitivities, bounded diagnostics and low aftercare value [4]. Extracellular matrix proteins play a significant role in the survival and metastasis of cancer cells. Thus, a group of extracellular matrix proteins, the Tenascins, recently came into the focus of cancer research [5–7]. Tenascins are a group of extracellular matrix glycoproteins that are expressed during the development of multicellular organisms and in pathological processes such as inflammation and tissue injury as well as tumor angiogenesis and metastasis [8]. Tenascin-C is a multi-domain disulfide-linked homohexamer consisting of 15 EGF-like domains followed by 15 fibronectin-type-III domains and a C-terminal fibrinogen domain [9]. The Tenascin protein family consists of four members (-X, -R, -C, -W), of which Tenascin-X and -R are also present in differentiated, healthy tissues. However, in adults Tenascin-C and -W are expressed only in pathophysiological conditions such as cancer development [5]. A significant expression of Tenascin-C can be measured in embryogenesis, at sites with a high cell turnover, such as stem cell niches and wound healing and inflammatory diseases, i.e. rheumatoid arthritis [6]. Several isoforms of Tenascin-C are formed by alternative splicing and it was reported that especially its large isoform plays a decisive role in the regulation of angiogenesis and in tumorigenesis of cancer [9–11]. The prognostic significance of Tenascin-C expression was investigated in several malignant diseases, such as squamous cell carcinoma of the head and neck, breast, colorectal and prostatic cancer [12–16]. Little is known regarding the expression and functional role of the large isoform of Tenascin-C in NSCLC, it is overexpressed in specimens of NSCLC and Parekh and colleagues described increased expression in patients with an early recurrence of disease [17–20]. In an analysis of endobronchial epithelial-lining fluid of NSCLC patients, Tenascin-C was significantly elevated and the authors suggested that it might be a potential diagnostic tool for early cancer detection [21]. Ishiwata and colleagues described a significant correlation of overall survival and elevated Tenascin-C serum values in a cohort of 63 NSCLC patients, but there was no association of clinical and pathological data and the molecule [22]. Moreover, Tenascin-C expression was investigated in COPD lung specimen and patients' serum with divergent results [23, 24].

We conducted the present study to determine the association between preoperatively sampled serum values of the large isoform of Tenascin-C and clinical and histopathological data and to evaluate its significance as a diagnostic and prognostic marker of resectable NSCLC patients.

## RESULTS

### Serum Tenascin-C levels in NSCLC patients and healthy controls

**Figure 1:**
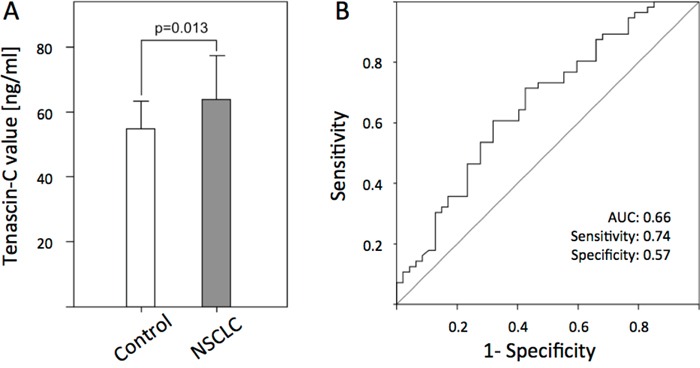
Serum concentration of Tenascin-C in healthy controls and non-small-cell bronchogenic carcinoma (NSCSC) patients **A.** Bars represent the mean of n= 76 healthy controls and n = 116 NSCLC patients as determined by ELISA (p<0.013). **B.** Receiver operating characteristic (ROC) curves of Tenascin-C for the diagnosis of NSCLC patients versus healthy control samples.

Tenascin-C concentration in NSCLC-patients was significantly elevated (n=103, mean 5.5 ng/ml, standard deviation (SD) ±3.8 ng/ml) compared to healthy blood donors (n=76, mean 6.3/ml, SD ±6.6ng/ml, p=0.013; Figure 1A). Receiver operating characteristic curves were used to establish the sensitivity-specificity relationship for Tenascin-C (1B). The AUC was 0.690. The cut-off level determined by the Youden index was 35.6 ng/ml. The sensitivity of Tenascin-C in detecting NSCLC was 74.4% at a specificity of 57.4% compared to the control group.

### Association of Tenascin-C serum concentrations and clinical and pathological parameter

Patients were divided into low level (<8.33 ng/ml) and high-level Tenascin-C groups (≥8.33 ng/ml). The correlation of clinico-pathological data with Tenascin-C using cross tabulation showed a significant association of increased Tenascin-C and tumor sizes (pT3 and pT4 compared to pT1 and pT2 p = 0.022) and the status of the lymph nodes (pN2 and pN3 versus pN1; p = 0.036), respectively. Clinical parameters such as age, sex, perioperative mortality (30 days), resection, nicotine abuse, asbestos exposure, neoadjuvant therapy and adjuvant therapy did not show any significant differences compared to controls. We found no significant correlation of Tenascin-C with the development of distant metastases; however, we only assessed a very small number (n=3) of metastasized patients (Table 1).

**Table 1:** Association of clinicopathological characteristics of NSCLC patients and Tenascin serum levels

		All (n)	Tenascin-C high (n and %)	Tenascin-C low (n and %)	Significance (p)
**Age (cut65)**	<65	43	18 (42%)	25 (58%)	
	≥ 65	60	25 (42%)	35 (58%)	1.000
**Sex**	Male	70	31 44%)	39 (58%)	
	Female	33	12 (36%)	21 (64%)	0.523
**Asbestos exposition**^†^	Yes	5	2 (40%)	3 (60%)	
	No	85	35 (41%)	50 (59%)	1.000
**Nicotine abuse**^†^	Yes	15	5 (33 %)	10 (67%)	
	No	76	32 (42%)	44 (58%)	0.579
**Neoadjuvant Therapy**^†^	Yes	82	32 (39%)	50 (61%)	
	No	11	6 (55%)	5 (45%)	0.347
**Tumor size**^†^	pT1+2	74	26 (35%)	48 (65%)	
	pT3+4	20	13 (65%)	7 (35%)	**0.022**
**Lymph nodes**^†^	pN0+N1	45	13 (29%)	32 (71%)	
	pN2+N3	50	26 (52%)	24 (48%)	**0.036**
**Distant metastases**^†^	M0	94	38 (40%)	56 (60%)	
	M1	8	4 (50%)	4 (50%)	1.000
**Grading**^†^	G1 and G2	41	16 (39%)	25 (61%)	
	G3	51	21 (41%)	30 (59%)	1.000
**Cell type**	Adeno	39	13	26	
	Sqamous Cell	37	21	16	
	Large Cell	12	6	6	0.115
**Resection**^†^	R0	68	25 (37%)	43 (63%)	
	R1	7	4 (57%)	3 (43%)	0.419
**30-day Mortality**^†^	Yes	10	3 (30%)	7 (70%)	
	No	91	39 (43%)	52 (57%)	0.516
**Adjuvant Therapy**^†^	Yes	42	17 (41%)	25 (59%)	
	No	51	21 (41%)	30 (59%)	1.000

† Due to the retrospective character of the study, numbers do not always add up to 103 patients.

### Kaplan-Meyer survival curve for recurrence-free survival in NSCLC patients

**Figure 2:**
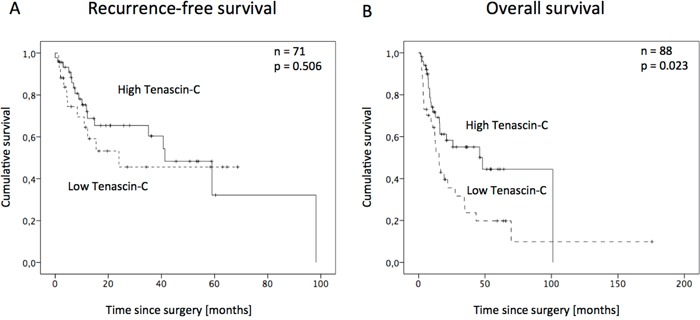
Kaplan-Meyer survival curve for recurrence-free survival **A.** and overall survival **B.** in NSCLC patients after surgery for Tenascin-C low- and high-level group.

Survival curves plotted by the Kaplan-Meier analysis (log-rank test) did not show a significant correlation of Tenascin-C-level and RFS (median calculated RFS 41.3 months and 24 months, respectively; p=0.328, Figure 2A). In contrast, prolonged OS was significantly associated with a lower Tenascin-C serum value (p=0.004, Figure 2B). Patients with concentrations under 8.33 ng/ml showed a median OS of 48.1 months, whereas those with concentrations over 8.33 ng/ml only survived for 15.4 months as a median.

### Multivariate analysis

In univariate survival analysis performed by cox-regression, a significant impact of clinic-pathological data such as tumor size, lymph node status, resection, grading and distant metastasis was shown (Table 2). While a significant factor in univariate analysis, Tenascin-C failed to be an independent prognosticator for survival in multivariate analysis. The analysis identified the resection status (R) and the tumor cell grading (G) as independent characteristics.

**Table 2:** Prognostic value of Tenascin-C expression for overall survival in univariate and multivariate analyses by Cox regression

	Univariate analysis			Multivariate analysis		
	HR	95%CI	*P*-value	HR	95%CI	*P*-value
Age	1.325	0.940/1.868	0.108	**-**	**-**	**-**
Sex	0.882	0.614/1.268	0.498	**-**	**-**	**-**
Tumor size(pT3/4 vs 1/2)	2.135	1.434/3.233	0.001	1.606	0.706/3.652	0.259
Lymph node metastases(pN2/3 vs 1/2)	1.850	1.301/2.630	0.001	2.098	0.901/4.885	0.086
Resection margin (R1/2 vs 0)	2.360	1.411/3.948	0.001	3.470	1.163/10.355	**0.026**
Grading (G3 vs 1/2)	1.600	1.129/2.269	0.008	2.853	1.310/6.213	**0.008**
Distant metastases (M1 vs M0)	2.806	1.220/6.457	0.015	1.105	0.218/5.600	0.904
Tenascin-C (low vs high)	2.374	1.301/4.321	0.005	1.434	0.669/3.074	0.354

(HR- hazard ratio; CI- confidence interval)

## DISCUSSION

The aim of the current study was to evaluate the potential of Tenascin-C serum quantification (large isoform) as a diagnostic and prognostic marker in the peripheral blood of NSCLC patients. Until now, only few groups have analyzed the functional role of Tenascin-C in NSCLC. The results showed that Tenascin-C serum levels were significantly elevated compared to the sera of healthy controls (p<0.001). The AUC showed an acceptable discriminatory power of Tenascin-C (AUC = 0.66; Figure 1B). The sensitivity (74%) and specificity (57%) of Tenascin-C was clearly inferior to the most frequently used tumor markers for NSCLC, SCCA, NES or CEA [25]. These data, which are similar to those of Ishiwata and colleagues, who did not find a significant difference between NSCLC patient sera and healthy controls, enabled us to conclude that Tenascin-C might not be a proper screening tool for the diagnosis of NSCLC [22]. Another aspect underlying this hypothesis is the potential overexpression of Tenascin-C in COPD patients and smoking, which are frequent comorbidities of NSCLC patients [23, 24, 26].

We furthermore investigated the correlation of Tenascin-C serum values and histopathological factors, which revealed a significant association of elevated Tenascin-C values with larger tumor sizes and lymph node metastasis (p=0.022 and p=0.036, respectively), indicating a relatively elevated Tenascin-C expression and release in higher tumor stages with a larger tumor burden. This is contradictory to the results of Ishiwata and colleagues, who revealed no association with tumor stage [22].

According to our findings, patients with higher Tenascin-C serum values have a shortened OS compared to the low Tenascin-C group. These correlations were already indicated by Parekh and colleagues, who had a small cohort of ten patients and found elevated Tenascin-C mRNA and protein expression in patients with early recurrence of the disease and later confirmed by Ishiwata and colleagues, who described a significant correlation in a group of 63 patients [19]. However, as the multivariate Cox regression analysis in the present study failed to reveal Tenascin-C as an independent prognostic parameter for overall survival (p=0.354), the role as a potential prognostic tumor marker for NSCLC patients remains inconclusive. However, data on serum levels were published in few studies only. Depending on the tumor type, the findings of Tenascin-C serum levels and linkage to clinical parameter are variable. For example, analysis of Tenascin-C serum levels in breast and ovarian cancer did not reveal any correlations to the overall-survival or clinical data [27, 28].

Several studies have investigated the expression of Tenascin-C in various solid tumors and tumor microenvironments and supposed a functional role in tumor progression, migration and the formation of metastases [6]. Interestingly, some researchers identified low Tenascin-C as a marker for a better prognosis for some diseases [29–31], but others described a contrary effect, a correlation of high Tenascin-C and a better prognosis [32, 33]. These results are not surprising, as Tenascin-C mediates complex remodeling of the tumor microarchitecture by close interactions between tumor cells and surrounding stromal cells. Expression of Tenascin-C is associated with intra-tumoral angiogenesis i.e. micro-vessel density and a pro-migratory influence on endothelial cell migration [22]. Tenascin-C expression was found in both, stromal and cancer cells and has a direct influence on epithelia-mesenchymal transition.

Another functional role of Tenascin-C is immune-modulatory; it inhibits the proliferation of blood lymphocytes *in vitro* and interferon-gamma production by tumor-infiltrating lymphocytes isolated from NSCLC specimens [19]. The results of these studies might reveal an association between Tenascin-C and a malignant biology, which results in an elevated shedding of Tenascin-C by matrix metalloproteinases (MMPs) such as MMP-13 [34]. Additionally, the shedding of the molecule might be caused by a transformation of the surrounding tumor environment. Both mechanisms, the regulation of the molecule's shedding, the migration into the tumor environment and even the access into the bloodstream through the endothelial barrier are understood only in part. However, the elevated expression of Tenascin C in tumor tissue and corresponding serum levels is in line with the well-established idea of a relationship between inflammation and cancer. But, as previously shown, the immune response during malignant progression and treatment resistance is a dynamic process and can even initiate pathways that are pro-tumorigenic [35]. The interactions of the immune system with tumor cells are of high complexity since several different types of cells and, in addition, sub-differentiations in dependency of the surrounding cytokine environment, are reported. The effects, how inflammation promotes tumor initiation, progression and treatment resistance are still poorly understood.

In summary, the detected Tenascin-C might be a useful prognostic parameter, which power might be increased by combination with others. It could reveal additional discriminatory or prognostic power additional to innovative staging parameter, such as circulating disseminated tumor cells status or other molecular markers together and to established systems, such as the TNM staging. The goal is to identify those patients, which might best profit from the available treatments to tailor an individual therapy for each patient.

Further prospective studies with larger patient cohorts are needed to evaluate the predictive potential of Tenascin-C serum levels. Moreover, the functional role of Tenascin-C in NSCLC is poorly understood and needs to be investigated in in vitro and in vivo analyses; to increase the knowledge of Tenascin-C associated tumor proliferation and metastatic invasion.

## MATERIALS AND METHODS

### Characteristics of patients and healthy controls

Blood sera of Caucasian 103 patients, aged between 31 and 82 years (median 65.5 years) with the diagnosis of NSCLC and Caucasian 76 healthy blood donors, aged between 31 and 79 (median 51 years), were included in this study. All patients were treated surgically between 1993 and 2010 in the Department of General, Visceral and Thoracic Surgery of the University Medical Center Hamburg-Eppendorf, Germany. All blood samples were obtained directly before surgery. All sera were processed at the latest after four hours [36]. Operation methods were oncological lobectomies, bilobectomies and pneumectomies with hilar and mediastinal lymphadenectomy.

All data including sex, histology, tumor size, lymph node metastasis, disease stage (UICC 7th edition) and follow-up data were obtained from a combination of clinical and pathological record reviews, from outpatient clinic medical records and communication with patients and their attending physicians, and from the cancer registry. Reliable follow-up data were available for 88 patients. Written consent for using the samples for research purposes was obtained from all patients prior to surgery or blood drawing.

The Ethics Committee of the Chamber of Physicians in Hamburg, Germany approved the study. The study was performed in accordance with the principles of the declaration of Helsinki (as revised in Seoul 2008) and REMARK criteria [37].

Disease-free survival (DFS) was calculated from the date of operation to the first detection of tumor recurrence, while overall survival (OS) was accordingly from the date of operation to the date of death or last follow-up. Patients who did not survive the first 30 days after surgery were excluded from the survival analysis. Median follow-up time of the patients included in the survival analysis was 12.2 months (range 0-176 months); median calculated OS was 27.6 months (95% CI 9.7-45.4 months). Four (6.8%) patients died within the first 30 days after surgery.

### Enzyme-linked immunosorbent assay for human Tenascin-C

The serum concentration of human Tenascin-C was quantified using a TNC-ELISA (ELISA (Human Tenascin-C Large Assay Kit - Immuno-Biological Laboratories Co., Ltd., Japan) according to the manufacturer's instructions.

In brief, a microtiter plate pre-coated with monoclonal mouse anti-human Tenascin-C antibody (4C8MS) was incubated with the serum of NSCLC patients and serum of healthy controls, respectively for 1h at 37°C. After washing, a HRP conjugated anti-Tenascin-C mouse antibody (4F10TT) specific for the EGF-like domain was added and incubated for 30 min at 4°C. Another washing step followed before the addition of Tetra-Methyl-Benzidine as the coloring agent (Chromogen) for 30 min at room temperature. The reaction was stopped by adding 1N H2SO4 solution. The absorbance was measured at 450 nm on an ELISA reader (Dynatech MR5000; Pegasus Scientific, Rockville, MD, USA).

In order to ensure that the immunoassay was suitable for measuring clinical serum samples, reproducibility and linearity were examined. The assay showed excellent linearity with serial dilutions and showed <10% coefficient of variation (CV) for the intra- and inter-assay variability studies.

### Statistical analysis

The statistical analysis was performed using SPSS Statistics for Windows (Version 20, SPSS Inc., Chicago Ill, USA). Interdependence between ELISA results and clinical data was calculated using Chi-square and Fisher's exact tests and displayed in cross tables. The cut-off level for Tenascin-C quantification was determined using the Youden-index. Group differences were calculated by the t-test, ANOVA; Mann-Whitney or Kruskal-Wallis test. Survival curves were plotted using the Kaplan-Meier method and analyzed using the log-rank test. All tests were two-sided and p-values less than 0.05 were considered statistically significant. All variables achieving a P value ≤ 0.05 were included in a multivariate Cox regression model for determining independent prognosticators of DFS and OS.

## References

[1] Jemal A, Center MM, DeSantis C, Ward EM (2010). Global patterns of cancer incidence and mortality rates and trends. Cancer Epidemiology Biomarkers & Prevention.

[2] Edge SB, Compton CC (2010). The American Joint Committee on Cancer: the 7th edition of the AJCC cancer staging manual and the future of TNM. Annals of surgical oncology.

[3] Kalia M (2014). Biomarkers for personalized oncology: recent advances and future challenges. Metabolism.

[4] Schneider J (2006). Tumor markers in detection of lung cancer. Advances in clinical chemistry.

[5] Yoshida T, Akatsuka T, Imanaka-Yoshida K (2015). Tenascin-C and integrins in cancer. Cell Adhesion & Migration.

[6] Giblin SP, Midwood KS (2015). Tenascin-C: form versus function. Cell adhesion & migration.

[7] Spenlé C, Saupe F, Midwood K, Burkel H, Noel G, Orend G (2015). Tenascin-C: exploitation and collateral damage in cancer management. Cell adhesion & migration.

[8] Orend G, Chiquet-Ehrismann R (2006). Tenascin-C induced signaling in cancer. Cancer letters.

[9] Pas J, Wyszko E, Rolle K, Rychlewski L, Nowak S, Zukiel R, Barciszewski J (2006). Analysis of structure and function of tenascin-C. The international journal of biochemistry & cell biology.

[10] Jones FS, Jones PL (2000). The tenascin family of ECM glycoproteins: structure, function, and regulation during embryonic development and tissue remodeling. Developmental dynamics.

[11] Tsunoda T, Inada H, Kalembeyi I, Imanaka-Yoshida K, Sakakibara M, Okada R, Katsuta K, Sakakura T, Majima Y, Yoshida T (2003). Involvement of large tenascin-C splice variants in breast cancer progression. The American journal of pathology.

[12] Borsi L, Carnemolla B, Nicolò G, Spina B, Tanara G, Zardi L (1992). Expression of different tenascin isoforms in normal, hyperplastic and neoplastic human breast tissues. International journal of cancer.

[13] Pauli C, Stieber P, Schmitt U, Andratschke M, Hoffmann K, Wollenberg B (2001). The significance of Tenascin-C serum level as tumor marker in squamous cell carcinoma of the head and neck. Anticancer research.

[14] Atula T, Hedstrom J, Finne P, Leivo I, Markkanen-Leppanen M, Haglund C (2003). Tenascin-C expression and its prognostic significance in oral and pharyngeal squamous cell carcinoma. Anticancer research.

[15] Hauptmann S, Zardi L, Siri A, Carnemolla B, Borsi L, Castellucci M, Klosterhalfen B, Hartung P, Weis J, Stöcker G (1995). Extracellular matrix proteins in colorectal carcinomas. Expression of tenascin and fibronectin isoforms. Laboratory investigation; a journal of technical methods and pathology.

[16] Ibrahim SN, Lightner VA, Ventimiglia JB, Ibrahim GK, Walther PJ, Bigner DD, Humphrey PA (1993). Tenascin expression in prostatic hyperplasia, intraepithelial neoplasia, and carcinoma. Human pathology.

[17] Kusagawa H, Onoda K, Namikawa S, Yada I, Okada A, Yoshida T, Sakakura T (1998). Expression and degeneration of tenascin-C in human lung cancers. British journal of cancer.

[18] Oyama F, Hirohashi S, Shimosato Y, Titani K, Sekiguchi K (1991). Qualitative and quantitative changes of human tenascin expression in transformed lung fibroblast and lung tumor tissues: comparison with fibronectin. Cancer research.

[19] Parekh K, Ramachandran S, Cooper J, Bigner D, Patterson A, Mohanakumar T (2005). Tenascin-C, over expressed in lung cancer down regulates effector functions of tumor infiltrating lymphocytes. Lung Cancer.

[20] Cai M, Onoda K, Takao M, Kyoko I-Y, Shimpo H, Yoshida T, Yada I (2002). Degradation of tenascin-C and activity of matrix metalloproteinase-2 are associated with tumor recurrence in early stage non-small cell lung cancer. Clinical cancer research.

[21] Kahn N, Meister M, Eberhardt R, Muley T, Schnabel PA, Bender C, Johannes M, Keitel D, Sültmann H, Herth FJ (2012). Early detection of lung cancer by molecular markers in endobronchial epithelial-lining fluid. Journal of Thoracic Oncology.

[22] Ishiwata T, Takahashi K, Shimanuki Y, Ohashi R, Cui R, Takahashi F, Shimizu K, Miura K, Fukuchi Y (2005). Serum tenascin-C as a potential predictive marker of angiogenesis in non-small cell lung cancer. Anticancer research.

[23] Munoz-Esquerre M, Huertas D, Escobar I, Lopez-Sanchez M, Penin R, Peinado V, Barbera JA, Molina-Molina M, Manresa F, Dorca J, Santos S (2015). Gene and Protein Expression of Fibronectin and Tenascin-C in Lung Samples from COPD Patients. Lung.

[24] Lofdahl M, Kaarteenaho R, Lappi-Blanco E, Tornling G, Skold MC (2011). Tenascin-C and alpha-smooth muscle actin positive cells are increased in the large airways in patients with COPD. Respir Res.

[25] Wang R, Wang G, Zhang N, Li X, Liu Y (2013). Clinical Evaluation and Cost-Effectiveness Analysis of Serum Tumor Markers in Lung Cancer. BioMed Research International.

[26] Karvonen HM, Lehtonen ST, Harju T, Sormunen RT, Lappi-Blanco E, Makinen JM, Laitakari K, Johnson S, Kaarteenaho RL (2013). Myofibroblast expression in airways and alveoli is affected by smoking and COPD. Respir Res.

[27] Didem T, Faruk T, Senem K, Derya D, Murat S, Murat G, Oznur K (2014). Clinical significance of serum tenascin-c levels in epithelial ovarian cancer. Tumor Biology.

[28] Tastekin D, Tas F, Karabulut S, Duranyildiz D, Serilmez M, Guveli M, Vatansever S (2014). Clinical significance of serum tenascin-C levels in breast cancer. Tumor Biology.

[29] Juhász A, Bárdos H, Répássy G, ádány R (2000). Characteristic distribution patterns of tenascin in laryngeal and hypopharyngeal cancers. The Laryngoscope.

[30] Jahkola T, Toivonen T, Virtanen I, von Smitten K, Nordling S, von Boguslawski K, Haglund C, Nevanlinna H, Blomqvist C (1998). Tenascin-C expression in invasion border of early breast cancer: a predictor of local and distant recurrence. British journal of cancer.

[31] Ishihara A, Yoshida T, Tamaki H, Sakakura T (1995). Tenascin expression in cancer cells and stroma of human breast cancer and its prognostic significance. Clinical cancer research.

[32] Iskaros BF, Tanaka KE, Hu X, Kadish AS, Steinberg JJ (1997). Morphologic pattern of tenascin as a diagnostic biomarker in colon cancer. Journal of surgical oncology.

[33] Pilch H, Schäffer U, Schlenger K, Lautz A, Tanner B, Höckel M, Knapstein P (1999). Expression of tenascin in human cervical cancer–association of tenascin expression with clinicopathological parameters. Gynecologic oncology.

[34] Hancox RA, Allen MD, Holliday DL, Edwards DR, Pennington CJ, Guttery DS, Shaw JA, Walker RA, Pringle JH, Jones JL (2009). Tumour-associated tenascin-C isoforms promote breast cancer cell invasion and growth by matrix metalloproteinase-dependent and independent mechanisms. Breast Cancer Res.

[35] Finn OJ (2008). Cancer immunology. N Engl J Med.

[36] Tuck MK, Chan DW, Chia D, Godwin AK, Grizzle WE, Krueger KE, Rom W, Sanda M, Sorbara L, Stass S (2008). Standard operating procedures for serum and plasma collection: early detection research network consensus statement standard operating procedure integration working group. Journal of proteome research.

[37] McShane LM, Altman DG, Sauerbrei W, Taube SE, Gion M, Clark GM (2005). REporting recommendations for tumour MARKer prognostic studies (REMARK). British journal of cancer.

